# Sepsis-Induced Cardiomyopathy and Cardiac Arrhythmias: Pathophysiology and Implications for Novel Therapeutic Approaches

**DOI:** 10.3390/biomedicines13112643

**Published:** 2025-10-28

**Authors:** Konstantinos Pamporis, Paschalis Karakasis, Antonia Pantelidaki, Panagiotis Antonios Goutis, Konstantinos Grigoriou, Panagiotis Theofilis, Athanasia Katsaouni, Michail Botis, Aikaterini-Eleftheria Karanikola, Nikias Milaras, Konstantinos Vlachos, Dimitrios Tsiachris, Constantinos Pantos, Iordanis Mourouzis

**Affiliations:** 1 Department of Pharmacology, University of Athens, 75 Mikras Asias Avenue, 11527 Goudi, Greece; antpantelidaki@gmail.com (A.P.); pangout04@gmail.com (P.A.G.); dinosgrigoriou@gmail.com (K.G.); athanasia.paraskevikatsaouni@gmail.com (A.K.); cpantos@med.uoa.gr (C.P.); imour@med.uoa.gr (I.M.); 2 First Cardiology Department, “Hippokration” General Hospital, School of Medicine, National and Kapodistrian University of Athens, 11528 Athens, Greece; panos.theofilis@hotmail.com (P.T.); mgmpotis94@gmail.com (M.B.); elinakaranikola@gmail.com (A.-E.K.); nikiasmilaras@gmail.com (N.M.); dtsiachris@yahoo.com (D.T.); 3 Second Department of Cardiology, General Hospital “Hippokration”, Aristotle University of Thessaloniki, 54642 Thessaloniki, Greece; pakar15@hotmail.com; 4 INSERM, CRCTB, U 1045, IHU Liryc, University of Bordeaux, F-33600 Pessac, France; vlachos.konstantinos7@gmail.com; 5 Cardiac Arrhythmia Department, INSERM, U 1045, CHU de Bordeaux, Avenue du Haut Lévêque, F-33604 Pessac, France

**Keywords:** sepsis, sepsis-induced cardiomyopathy, arrhythmias, atrial fibrillation, pathophysiology, treatment

## Abstract

In the context of multi-organ involvement in sepsis, cardiac toxicity is manifested as sepsis-induced cardiomyopathy (SICM). To date, no unified SICM definition exists, though a left ventricular ejection fraction ≤ 50% and/or an absolute drop ≥ 10% from baseline are the most widely accepted components. Several molecular pathways have been associated with SICM, including (i) pro-inflammatory mediator-induced cardiac depression; (ii) sarcolemmal membrane dysfunction; (iii) autonomic nervous system (ANS) imbalance; (iv) blunted cardiovascular response to catecholamines; (v) dysfunctional intracellular calcium handling; (vi) mitochondrial dysfunction; (vii) metabolic reprogramming; and (viii) disturbed endothelial and microcirculatory function. Atrial and ventricular arrhythmias—particularly atrial fibrillation—commonly complicate disease management and are associated with adverse outcomes. Key mechanisms outlining sepsis-induced arrhythmogenesis are (i) inflammation; (ii) electrolyte imbalances; (iii) myocardial ischemia; (iv) QT prolongation/dispersion; (v) adrenergic overactivation; (vi) calcium mishandling; and (vii) fever-induced arrhythmogenesis in Brugada. Established therapeutic approaches include prompt treatment with antibiotics, hemodynamic optimization, and/or selective use of beta-blockers. Furthermore, several molecules are currently being investigated targeting numerous pathways activated in sepsis. Vitamin C, ginsenoside Rc, Schistosoma Japonicum cystatin, and gasmerdin-D inhibitor Y2 exert anti-inflammatory actions, while melatonin and α-ketoglutarate regulate mitochondrial homeostasis. Triiodothyronine targets microcirculatory optimization and regulates protective pathways against stress-related cell death. Engineered exosomes may facilitate targeted drug delivery, inflammatory response modulation, and activation of pathways related to cell survival, while sodium octanoate exhibits anti-inflammatory actions coupled with improved energy metabolism. Finally, gene-regulating therapies aiming at inflammatory response optimization have also been proposed and are currently under development. Future research should aim to standardize the SICM definition, translate emerging therapeutics into clinical practice, identify novel molecular targets, and implement personalized treatment strategies for SICM.

## 1. Introduction

Sepsis and septic shock constitute major causes of morbidity and mortality worldwide with increasing incidence trends [1]. Sepsis-induced cardiomyopathy (SICM) constitutes a manifestation of cardiac dysfunction due to sepsis [2]. During SICM, several pathophysiological disturbances occur, with the heart being affected as the final target [3]. It is estimated that SICM affects 10–70% of septic patients, with this large variability being mainly attributed to significant differences in SICM definition across the literature [4,5].

Despite not being formally integrated within the SICM definition, cardiac arrhythmias frequently complicate the clinical course of sepsis [6]. Several similar pathways are involved in the incidence of both SICM and sepsis-induced arrhythmias, though additional etiologies frequently contribute to sepsis-induced arrhythmogenesis. Among sepsis-induced arrhythmias, the most prevalent is atrial fibrillation (AF); nevertheless, any atrial or ventricular arrhythmia may occur.

Despite extensive research on sepsis, therapeutic interest in SICM has emerged only recently, with most evidence derived from preclinical models. The present narrative review sought to summarize the existing evidence on the pathophysiology of SICM and sepsis-induced arrhythmias, with a parallel reference of emerging therapeutics in the field.

## 2. Methods

MEDLINE (Pubmed) and Scopus were searched from inception until 20 July 2025 using the following key terms: “sepsis” AND (“cardiomyopathy” OR “arrhythmias”). For each term, synonyms were combined using OR. For MEDLINE, MeSH terms were also searched. Eligible articles were clinical and preclinical studies, narrative/scoping/systematic reviews, and guidelines. The references of included studies were also scrutinized. Following study selection, the evidence was grouped by topic (diagnosis, pathophysiology, current management, novel interventions) and synthesized in a non-systematic, qualitative manner to produce a narrative literature review. Where possible, greater interpretative weight was given to higher levels of evidence (e.g., randomized or large prospective studies, systematic reviews, and consensus guidelines), while preclinical and smaller observational studies were used to support mechanistic or exploratory insights.

## 3. Sepsis-Induced Cardiomyopathy

### 3.1. Epidemiology and Definition

In the context of sepsis, myocardial dysfunction is defined as SICM [2]. The prevalence of SICM varies considerably (10–70%) depending on the definition and population used in each study [4,5], the diagnostic methods, and the temporal relationship between sepsis onset and diagnosis (Table 1). Interestingly, SICM prevalence gradually increases after sepsis onset [7,8] based on an observational cohort of 67 patients, where SICM prevalence increased from 18% within six hours to 60% within 72 h [8].

**Figure 1:**
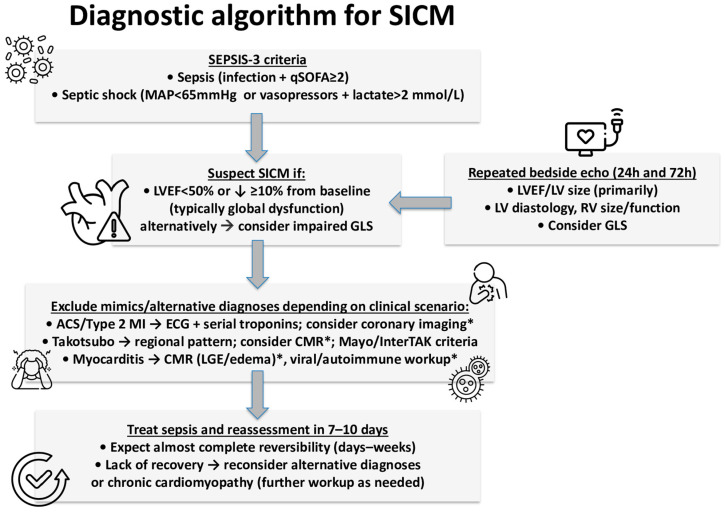
Diagnostic approach to sepsis-induced cardiomyopathy (SICM). The algorithm outlines the sequential steps for identifying SICM in septic patients—starting with SEPSIS-3 criteria, echocardiographic suspicion based on reduced LVEF or GLS, exclusion of alternative diagnoses (Ischemia, Takotsubo, myocarditis), and reassessment after sepsis treatment to confirm reversibility. Specialized/advanced diagnostic examinations are marked with asterisk (*) and may be performed depending on the clinical setting (not routinely). Abbreviations: ACS, acute coronary syndrome; CMR, cardiac magnetic resonance; ECG, electrocardiogram; GLS, global longitudinal strain; InterTAK, International Takotsubo Diagnostic Criteria; LGE, late gadolinium enhancement; LV, left ventricle; LVEF, left ventricular ejection fraction; MAP, mean arterial pressure; MI, myocardial infarction; qSOFA, Quick Sequential Organ Failure Assessment; RV, right ventricle; SICM, sepsis-induced cardiomyopathy.

During SICM, the systolic [8,13,29,30,31] and/or diastolic [29,32,33,34,35] function of the left ventricle (LV) may be affected alongside right ventricular (RV) dysfunction [11,36,37,38,39]. Around 30% of septic patients present a reversible LV systolic dysfunction, which is manifested with hypokinesis and reductions in LV ejection fraction (LVEF) [30]. Hence, most researchers propose an LVEF ≤ (40–50%) or a reduction ≥ 10% in pre-existing systolic dysfunction as the main component of SICM definition. Furthermore, myocardial dysfunction is typically characterized by acute onset sand reversibility (usually within 7–10 days) and is unrelated to coronary artery disease (including acute coronary syndromes) [3,40,41,42,43] or mimickers like myocarditis, stress-induced cardiomyopathy, or other cardiomyopathies (Figure 1). Despite the commonly encountered RV dysfunction (frequently >50%) [11,38,39], this component is often omitted from the definition due to concomitant factors affecting RV function [40], like mechanical ventilation and adult respiratory distress syndrome [44]. Finally, LV diastolic dysfunction complicates sepsis in approximately 50% [45].

The prognostic implications of SICM remain unclear, and their variability is partly attributed to the different methods and definitions used to delineate myocardial dysfunction. Studies measuring LVEF have demonstrated negative (lower LVEF associated with improved survival) [46,47] and neutral [29,30,31,45] associations. Of interest, Chotalia et al. reported that patients with LVEF > 70% had worse survival, probably due to profound reduction of systemic vascular resistance and/or catecholamine hypersecretion [48]. In contrarst, studies using global longitudinal strain (GLS) have reported positive associations with increased mortality [49,50,51,52]. Interestingly, GLS constitutes a systolic marker that is affected earlier compared to LVEF [7] while also being less preload- and afterload-dependent. Collectively, these characteristics render GLS a more objective and accurate marker of systolic function compared to LVEF [12,53]. A meta-analysis of studies that used the systolic S’ wave to evaluate systolic dysfunction [54] found no pertinent associations. Finally, myocardial work evaluated via echocardiography was recently associated with mortality in a cohort of septic patients [55]; nevertheless, its use might be limited due to time-consuming evaluation and limited availability.

Diastolic dysfunction has also been associated with increased mortality (up to 80%) in certain studies [34,45,56]. However, diastolic evaluation is not uniformly performed, and not all diastolic markers present the same accuracy. For example, the meta-analysis from Sanfilippo et al. reported that the lateral e′ wave was better correlated with mortality compared to the diastolic septal e′ [34]. Regarding the RV, almost half of septic patients present some degree of RV dysfunction [11,38,39] with an estimated 30–60% increase in mortality [38,57,58]. Of note, prognostic associations have only been reported for systolic markers other than RV free wall strain, which did not demonstrate prognostic relevance in two prospective cohorts [55,59].

Regarding the risk stratification of septic patients, factors found to be associated with SICM are male sex, younger age, higher lactate, and previous heart failure, while the infectious site seems to be unrelated [14,43]. Several biomarkers could also provide useful diagnostic and prognostic information, including Suppression of Tumorigenicity 2 (ST2), galectin-3, and endothelin-1 (ET-1). Indeed, ST2 has been associated with mortality in males with SICM [60] and in critically ill septic patients [61], including patients with heart failure [62]. Galectin-3 could predict poor outcomes in sepsis [63] and in patients with inflammatory cardiomyopathies [64], while high ET-1 has also been correlated with increased morbidity and mortality through microvascular dysfunction [65,66]. Nevertheless, the existing studies are observational with small sample sizes and lack of external validation.

### 3.2. Pathophysiological Mechanisms of SICM

**Figure 2:**
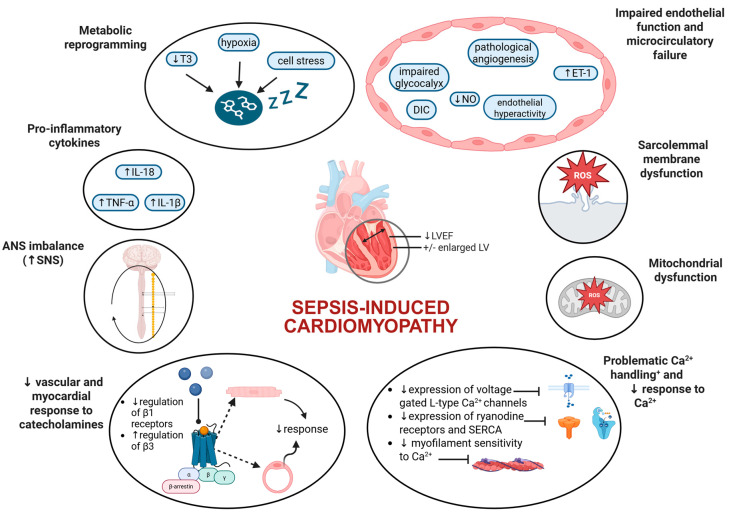
Pathophysiological mechanisms of sepsis-induced cardiomyopathy (SICM). Abbreviations: ANS, autonomic nervous system; DIC, disseminated intravascular coagulation; ET-1, endothelin-1; IL-1β, interleukin-1β; LV, left ventricle; LVEF, left ventricular ejection fraction; NO, nitric oxide; SERCA, sarco-plasmic/endoplasmic reticulum Ca^2+^-ATPase; SNS, sympathetic nervous system; TNF-α, tumor necrosis factor-α.

A complex interplay has been proposed for SICM development with an interaction between host-related (immunologic, genetic, epigenetic susceptibility) and environmental factors (pathogens, iatrogenic causes). Upon histological examination, SICM is characterized by tissue infiltration from inflammatory cells coupled with contraction band necrosis, interstitial fibrosis, and edema, all of which mediate cardiotoxicity [67,68]. The pathophysiological mechanisms leading to SICM are described below (Figure 2):

#### 3.2.1. Myocardial Depression Due to Inflammation

During sepsis, several molecules are recognized by immune cells, mainly via toll-like receptors (TLRs), leading to activation of various intracellular pathways, including nuclear factor-kB (NF-kB) and mitogen-activated protein kinase (MAPK) [69,70]. In turn, these pathways lead to the production of pro-inflammatory cytokines that further orchestrate the immunological response. Multiple preclinical and clinical studies suggest that myocardial depression may occur due to direct toxicity from cytokines, including tumor necrosis factor-α (TNF-α), interleukin (IL) 1-β [71,72,73,74,75,76], and endothelin (ET)-1 [77,78]. Moreover, in a murine model of lipopolysaccharide (LPS)-induced sepsis, TLR-4-deficient mice demonstrated better myocardial function compared to controls [75].

Furthermore, in two very small preclinical studies, TNF-α infusions in dogs have been associated with myocardial dysfunction within the first two days after the infusion [79,80], while lower IL-32 levels have been linked to better outcomes in septic patients [81]. However, the latter study was observational without multivariable adjustments, while authors measured IL-32 only during admission without differentiating between IL-32 isoforms. Exosomes released by infected macrophages may also be implicated in SICM (via perpetuation of inflammation) considering that their targeted blockade has been associated with improved myocardial function and better outcomes in mice [82]. Accordingly, upregulation of the anti-inflammatory pathway of IL-17 and interferon (IFN) have been associated with improved survival in 74 patients with SICM [74]. Nonetheless, this was a small monocentric study, whose results remain to be externally validated.

#### 3.2.2. Membrane Dysfunction and Attenuated β-Adrenergic Response

Impairment of the sarcolemmal membrane during sepsis is mediated through structural and functional changes in lipids (mainly cholesterol reduction) [83] and proteins [84] due to oxidative stress [85]. Subsequent consequences are the increased permeability of the cell membrane leading to interstitial edema as well as decreased response to catecholamines due to functional alterations in β-adrenergic signaling [86]. Additionally, reduced β1-adrenergic receptor expression [87] and activity is coupled with attenuation in the downstream signaling pathway [87,88] or even upregulation of the β3-adrenergic receptor that exhibits a negative inotropic response [89].

#### 3.2.3. Autonomic Nervous System (ANS) Imbalance

During sepsis, various dysregulations lead to sympathetic nervous system (SNS) overactivation, either due to endogenous activation (triggered by reduced systemic vascular resistance and hypovolemia) or due to iatrogenic factors (sympathomimetic medications). In turn, SNS overactivation leads to a depressive (inhibiting) response of G-coupled proteins [90,91], similarly to stress-induced cardiomyopathy [92,93]. Interestingly, the degree of histopathological myocardial disturbances has been associated with larger doses and duration of catecholamine administration in a cohort of 20 patients that died from septic shock [68]. However, the lack of control group, the small sample size, and the difficulty of establishing a temporal relationship between histological changes and catecholamine administration should be considered. Inappropriate SNS activation may also lead to dysregulated calcium homeostasis [94], oxidative stress [95], Na^+^/K^+^-ATPase pump dysfunction [96], cell death, and sepsis-induced arrhythmogenesis via triggered activity [97].

#### 3.2.4. Dysregulated Calcium Handling

Calcium mishandling [98,99] and reduced myocardial sensitivity to calcium [88,98,100,101,102] have long been observed in sepsis. These effects are mainly attributed to attenuated calcium currents due to reduced expression of the voltage-gated L-type calcium channels [103] and ryanodine receptors [94]. Additionally, TNF-α may also promote sarcoplasmic reticulum calcium ATPase (SERCA) dysfunction, thus inhibiting calcium reuptake [104]. Since calcium plays a key role in electromechanical coupling, these changes result in negative inotropy.

Interestingly, in a murine model of sepsis, L-type calcium current modulation via the cardiac chaperone melusin led to improvements in ventricular contractility [105], further supporting this hypothesis. In another murine model of SICM, prevention of SERCA downregulation was associated with significant improvements in myocardial function [106]. Improvements in cardiac contractility have been associated with better intracellular calcium handling in preclinical murine models [107]. Although these studies were conducted in different settings using various mouse strains and sepsis induction methods, their consistent findings support the role of calcium in the pathophysiology of SICM. Finally, increased intracellular calcium shortens the atrial refractory period and may precipitate arrhythmogenesis via triggered activity [108].

#### 3.2.5. Mitochondrial Dysfunction

In sepsis, mitochondrial dysfunction is mainly characterized by structural and functional alterations coupled with impairment of mitochondrial membrane potential [109]. Accordingly, the degree of myocardial dysfunction in SICM has been closely linked to the extent of oxidative stress generated by energy depletion [110]. Functional abnormalities include downregulation of genes involved in the synthesis of critical proteins, a finding observed both in preclinical and human studies [111]. Additional preclinical evidence support that sepsis may also reduce mitochondrial renewal capacity, leading to a decreased mitochondrial count [101,112,113]. Furthermore, in a male mouse model of SICM, blockage of carbohydrate catabolism was observed due to decreased pyruvate dehydrogenase (PDH) activity as a result of increased PDH kinase isozyme 4 (PDK4) concentration [114]. Similar findings were reported in another study, which demonstrated a sex-specific PDK4-mediated PDH inactivation occurring in male but not in female mice, possibly due to the protective effects of estradiol [115]. Collectively, these perturbations affect oxidative phosphorylation, leading to decreased adenosine triphosphate (ATP)-producing capacity and increased production of toxic reactive oxygen species (ROS) and oxidative stress [110].

#### 3.2.6. Metabolic Reprogramming

Metabolic reprogramming constitutes a preservatory response to tissue hypoxia, cell stress, and increased metabolic demands. In an effort to reduce demands, the myocardium enters a hibernation state, preventing further oxidative stress at the expense of reduced contractility [116,117]. This process is mediated through modified gene expression in several organs and tissues, including the heart [118,119], ultimately preventing ATP depletion and subsequent cell death [120]. It should be noted, however, that these data arise from small-scale animal studies with unclear extrapolation in humans. Additionally, sepsis causes neurohormonal disturbances through central dysregulation of the hypothalamic–pituitary axis and altered peripheral thyroid hormone metabolism, leading to reduced triiodothyronine (T3) and thyroxine (T4) levels, which underlie the development of euthyroid sick syndrome [119,121,122,123].

#### 3.2.7. Impaired Endothelial Function and Microcirculatory Failure

Impaired endothelial function [124] and microcirculatory failure [125,126] constitute principal pathophysiological disturbances in sepsis [127]. Severe sepsis and septic shock have a direct impact on microcirculation, reducing vessel density and modifying vascular perfusion via the vasa vasorum, observations originating from both preclinical [128] and clinical data [129]. Interestingly, the extent of microcirculatory dysfunction has been associated with worse clinical outcomes in experimental models [130,131,132] and in septic patients [129,133,134,135,136,137]. Furthermore, microcirculatory failure occurs despite the widespread use of interventions targeting macrocirculatory stabilization [122,138,139,140,141]. Also, clinical evidence in septic patients suggests that microcirculatory stabilization via intravenous fluids is not linearly associated with established hemodynamic markers, including arterial pressure [142] and stroke volume [143]. Similar findings have been observed after the administration of vasoactive substances [141,144]. Hence, different mechanisms are implicated in microcirculatory and macrocirculatory homeostasis.

The following mechanisms outline microcirculatory failure: (i) low oxygen partial pressure with subsequent release of hypoxia-induced toxic molecules; (ii) reduced concentration of antioxidants [145]; (iii) reduced nitric oxide (NO) production within microvasculature [146]; (iv) endothelial hyperreactivity to vessel tone changes [147]; (v) blunted interaction between endothelium and circulating cells (132); (vi) reduced function of endothelial glycocalyx due to cell edema and apoptosis [148,149]; (vii) formation of microthrombi and activation of circulating cell remnants [150]; (viii) pathological angiogenesis [151]; (ix) prostanoids produced by cyclooxygenase-2 activation leading to microvascular dysfunction in coronary circulation [152]; (x) ET-1 overexpression leading to platelet activation and vasoconstriction, which is also associated to the development of severe SICM [153]; (xi) microthrombi formation due to disseminated intravascular coagulation [154,155], partly associated with the formation of neutrophil extracellular traps [156]; and (xii) myocardial edema due to cytokine-induced microvascular permeability [157] and reduced lymphatic drainage [158]. In turn, myocardial edema leads to reduced nutrient and oxygen delivery, coupled with reduced ventricular compliance [159,160,161].

## 4. Sepsis-Induced Cardiac Arrhythmias

Despite not being formally included in the SICM definition, atrial and ventricular arrhythmias complicating sepsis could also be perceived as a form of cardiac toxicity. Arrhythmias may develop in septic patients regardless of a relevant history of cardiac arrhythmia or pre-existing cardiomyopathy. Nevertheless, pre-existing pathologies could precipitate their incidence. Indicatively, patients with atrial cardiomyopathy may be prone to atrial arrhythmias (especially AF) due to complex electroanatomical and functional remodeling [162,163], facilitating re-entry and triggered activity [164].

The most incident arrhythmia in sepsis is AF, and its occurrence is associated with poor outcomes. Interestingly, sepsis is associated with an approximately six-fold increased AF risk [6] and constitutes around 20% of the causes of “secondary” AF [165]. Interestingly, the more profound the septic syndrome, the higher the probability of developing AF [166,167]. Acute AF management includes anticoagulation and rate or rhythm control selected on an individual basis [168]. Nevertheless, questions remain in the outpatient management. Recent evidence supports that catheter ablation is more effective when performed within one year of AF diagnosis [169], including in young individuals [170]. However, it is unclear whether this evidence should be universally applied in sepsis-induced AF, especially considering that patients with “secondary” AF are frequently excluded from research studies. Finally, septic patients may be particularly prone to asymptomatic AF [171,172], and artificial intelligence (AI)-enabled systems could further facilitate early detection and prompt therapeutic management [173].

**Figure 3:**
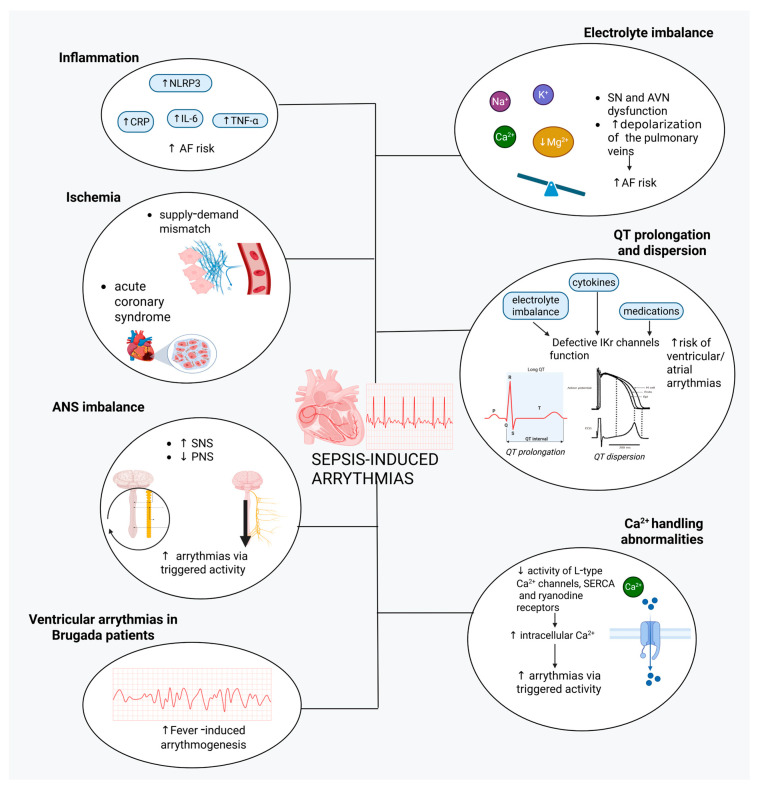
Pathophysiological mechanisms of sepsis-induced cardiac arrhythmias. Abbreviations: AF, atrial fibrillation; ANS, autonomic nervous system; AVN, atrioventricular node; CRP, c-reactive protein; IKr, rapid K^+^ current; IL-6, interleukin-6; NLRP3, NOD-like receptor family pyrin domain containing 3 of the NLRP3 inflammasome; PNS, parasympathetic nervous system; SERCA, sarcoplasmic/endoplasmic reticulum Ca^2+^-ATPase; SN, sinus node; SNS, sympathetic nervous system; TNF-α, tumor necrosis factor-α.

In addition to AF, other forms of atrial and ventricular arrhythmias may also occur. Of note, septic patients are prone to cardiac arrest, and cardiac arrhythmias may frequently be among the principal causes [174,175]. The etiological substrate of sepsis-induced arrhythmias is multifactorial, and besides the previously described ANS imbalance and calcium mishandling, further contributing factors are outlined below (Figure 3).

### 4.1. Inflammation

Inflammatory markers including C-reactive protein (CRP), IL-6, and TNF have been associated with increased incidence of new-onset AF [176,177]. The binding of CRP to phosphocholine can cause direct myocardial damage via membrane dysfunction and inhibition of the sodium and calcium channels, ultimately promoting arrhythmogenesis [178]. Interestingly, myocarditis with active myocardial inflammation has been identified as an important cause of non-ischemic sudden cardiac death (9% in a group of 453 patients) [179]. Despite being unable to clearly demonstrate an arrhythmic cause of death, this study reinforces the interplay between inflammation and arrhythmogenesis.

Inflammasome signaling is also crucial, especially nucleotide-binding domain, leucine-rich repeat-containing receptor pyrin domain containing 3 (NLRP3) [180]. During cardiomyocyte damage and inflammation, intracellular activation of innate immune pathways via NLRP perpetuates tissue-specific inflammation within the heart via IL-1β and IL-18 secretion [181]. In turn, cytokines promote immune cell migration within atrial and ventricular tissue. Of note, experimental models suggest that genetically-modified mice that highly express NLRP3 also exhibit higher counts of spontaneous atrial contractions and inducible AF [182]. Consequently, this complex interplay between cardiomyocytes and immunity eventually increases AF incidence and perpetuation [183,184,185].

### 4.2. Electrolyte Abnormalities

Electrolyte abnormalities are frequently encountered during sepsis either due to patient- and sepsis-related factors or due to iatrogenic interventions. For example, changes in pH and adrenergic activity are directly associated with potassium and/or magnesium homeostasis, whereas several medications may induce or exacerbate electrolyte abnormalities. In turn, electrolyte disturbances induce or facilitate arrhythmias even in normal hearts, more so in pathological substrates [186]. Imbalances in sodium and potassium have been associated with sinus and atrial node dysfunction as well as increased ectopy in pulmonary veins [187,188]. In turn, ectopic activity significantly increases AF risk. Hypokalemia constitutes the most prevalent electrolyte abnormality in sepsis and leads to decreased action potential velocity, increased cardiac excitability, as well as increased automaticity and early afterdepolarizations, all of which contribute to arrhythmogenesis [186,189]. Magnesium interacts with potassium abnormalities, exhibits modulating actions in potassium channels, and may ultimately increase the risk for AF [190]. Disturbances in calcium homeostasis, particularly hypocalcemia, have also been associated with increased arrhythmogenesis [191,192].

### 4.3. Myocardial Ischemia

Myocardial ischemia may occur in sepsis either due to type supply–demand mismatch (type 2 myocardial infarction [MI]) [193] or due to an acute coronary syndrome [194], the latter being associated with a further increased mortality [195]. Epidemiological evidence suggests that sepsis is among the most commonly encountered non-cardiac diseases associated with acute MI. Indicatively, in a cohort of 637 hospitalized patients with acute MI, 96/637 (15%) were initially admitted for non-cardiac causes, and 59/96 (61%) had an initial diagnosis of sepsis [196].

Regardless of the underlying cause, ischemia-mediated arrhythmogenesis is driven by ATP deficiency, acidosis due to anaerobic metabolism, and potassium disturbances [197]. Ischemia shortens the duration of the action potential via (i) activation of I_Katp_ channels leading to outward potassium current; (ii) inhibition of I_K1_ channels leading to more positive resting membrane potential; (iii) intracellular calcium abnormalities; (iv) attenuated electrical conduction via gap junctions; and (v) QT prolongation [198]. Consequently, ischemia promotes arrhythmias mainly via triggered activity and less frequently via functional re-entry [199].

### 4.4. QT Prolongation and Dispersion

QT prolongation is also common during sepsis, largely driven by acquired long QT syndrome. QT interval represents the phase of ventricular depolarization and repolarization, and its prolongation is usually caused by defective I_Kr_ potassium channels [200]. Several factors contribute to QT prolongation, including sepsis itself, electrolyte abnormalities, and QT-prolonging medications [201]. In a small prospective cohort of 41 patients with acute infections, corrected QT (QTc) was significantly prolonged and correlated with CRP and cytokine levels [175]. Interestingly, the expression of KCNJ2 potassium channels was inversely associated with inflammatory markers, suggesting a downregulation induced by inflammation itself [175]. However, ventricular tissues were available only in seven patients, and thus the results on channel expression are prone to random error. QT prolongation increases the risk of atrial and ventricular arrhythmias induced mainly via triggered activity (early afterdepolarizations) during phase 3, while prolonged QT has been independently associated with mortality in a retrospective multicenter cohort of 1024 septic patients [202]. Additionally, QT dispersion constitutes another proarrhythmic risk factor. Indeed, inhomogeneous repolarization creates unidirectional blocks and zones of slow conduction, facilitating reentry and/or torsade de pointes. The ECG marker Tpeak to Tend duration (Tp-e)/QT has been associated with repolarization dispersion. In turn, prospective observational data from 625 patients (201 with sepsis) suggest that sepsis is associated with higher Tp-e/QT, which also constitutes a prognostic marker of increased mortality [203]. Besides QTc, murine models suggest that sepsis may also perturbate signal propagation due to dysfunctional gap junctions [204,205].

### 4.5. Fever-Induced Arrhythmias in Brugada Patients

Although they represent a minority, septic patients with Brugada syndrome are at particularly high risk of arrhythmias, primarily triggered by fever. Observational evidence suggests that fever increases the risk of inducing type 1 Brugada pattern by almost 20 times [206] due to high body temperatures that reduce sodium channel activity [207,208], ultimately increasing proarrhythmia. Of note, catheter ablation has demonstrated a significant benefit in symptomatic Brugada patients and may be considered in further management [209].

## 5. SICM Management and Novel Therapeutic Agents

The current mainstay of SICM management lies in initial hemodynamic stabilization with fluids (coupled with vasopressors when mandated) and treatment with medications (mainly antibiotics) to reduce the microbial burden [138]. If myocardial dysfunction persists, dobutamine is advised [138], with ambiguous survival benefits [210,211,212,213,214,215]. An ongoing randomized controlled trial (RCT) (NCT04166331) is awaited to shed more light on the effectiveness of dobutamine in sepsis. Levosimendan, acting via calcium sensitization, has not demonstrated any significant benefit [216,217,218,219] and may also aggravate hypotension due to further vasodilation. Additional therapeutic strategies have been proposed, such as the use of β-blockers in patients with persistent tachycardia despite initial resuscitation [220], which may offer a benefit but have not been shown to reduce mortality [221]. Corticosteroids also remains controversial [222]. Finally, cytokine-targeted therapies have been tested in RCTs with overall negative results. Anti-TNF strategies efficiently lowered TNF-α levels with no clinical benefit [223], while Anakinra (IL-1 receptor antagonist) has only proved efficacious in a limited number of patients with macrophage activation syndrome [224]. IL-6 receptor blockade has no supportive evidence in sepsis.

Interestingly, recent evidence suggests that there is no “one-size fits all” when treating sepsis [225,226]. Indeed, in a cohort of 360 patients, five different hemodynamic clusters were identified: well-resuscitated patients without LV systolic dysfunction, RV failure or fluid responsiveness (16.9%; 7-day mortality: 9.8%), patients with LV systolic dysfunction (17.7%; 7-day mortality: 32.8%), patients with hyperkinetic profile (23.3%; 7-day mortality: 8.3%), patients with RV failure (22.5%; 7-day mortality: 27.2%), and patients with persistent hypovolemia (19.4%; 7-day mortality: 23.2%) [227]. Of note, patients with LV dysfunction had higher mortality compared to patients with hyperkinetic LV (32.8% vs. 8.3%). Nevertheless, these patients also presented higher norepinephrine use, and hence, the degree of norepinephrine contribution to cardiac dysfunction (due to increased afterload) is unknown. Consequently, cardiovascular-targeted therapies should be individualized according to each patient’s phenotype. In this direction, non-invasive monitoring modalities like thoracic bioimpedance/bioreactance and Doppler-based methods provide continuous measurements of cardiac output without the need for arterial catheterization [228,229]. The following treatments are currently under investigation (Table 2).

- **Vitamin C** has been explored given its antioxidant and anti-inflammatory effect [230,231]; however, most clinical trials had negative results [232]. In contrast, only one study of 127 patients has provided evidence that septic patients presenting with an overt inflammatory response might benefit from vitamin C [233]. Additionally, a propensity score-matched analysis of 166 patients reported that vitamin C was associated with reduced use of vasopressors and improvement of clinical and laboratory markers [257]. Importantly, the time to therapy initiation was a significant effect modifier, since early administration (within 2 h) was associated with greater vasopressor weaning and lower mortality. However, given the retrospective design of both studies and the small sample sizes, these results should be interpreted with caution.
- **Melatonin** has been tested in several animal models of SICM. Melatonin exerts its beneficial effects mainly through the regulation of mitochondrial homeostasis. Macrophage-stimulating 1 (Mst1) overexpression has been associated with mitochondrial apoptosis, while melatonin reduces Mst1 expression in mice with SICM [234]. Melatonin regulates the JAK2/STAT3 pathway [235,236,237] and leads to elevated inducible NO synthase activity [238] providing vasodilatory effects. Furthermore, melatonin exerts several anti-inflammatory properties via the suppression of the hypoxia-inducible factor and the nuclear factor erythroid-2 related factor 2, alongside activation of the phosphatidylinositol 3–kinase (PI3K)/Akt signaling pathway [239,258,259], which could counteract myocardial depression due to inflammation. Collectively, all these effects coupled with a favorable safety profile render melatonin an attractive therapy in SICM. Nevertheless, clinical data is not available, and its efficacy in humans remains unknown.
- Engineered **exosomes** provide vehicles able to transfer specific molecules to targeted sites, acting via the three main mechanisms. (a) Direct and targeted drug delivery in specific tissues [260], including microRNAs [261], for example, delivery of MiR21-loaded exosomes to cardiomyocytes, produced significant anti-apoptotic effects and reduction of myocardial inflammation in a murine model of reperfusion injury [240], while exosomes containing miR-126 were associated with reduced expression of adhesion molecules in septic mice [262]. (b) Modulation of the inflammatory response [263] is based on evidence that exosomes can attenuate the TNF-a and IL-6 pathways even further when compared to established anti-inflammatory treatments [241]. (c) Enhancement of protective and reparatory pathways maintains cell survival [242,243]. It should be noted, though, that most evidence for exosomes arises from preclinical animal studies in MI without representation of SICM models. Small studies in humans have also been performed in various clinical settings [244,245,246] with promising results thus far. Hence, preclinical SICM models and large-scale human studies are lacking.
- Schistosoma japonicum-produced cystatin (**Sj-Cys**) is a cystatin originating from the trematode Schistosoma japonicum. During SICM, its use in a mouse model of cecal ligation and puncture (CLP)-induced sepsis was associated with several improvements in biomarkers and histological evidence of inflammation [247]. Sj-Cys-treated mice demonstrated reduced levels of cardiac troponin and natriuretic peptides as well reduced infiltration of inflammatory cells within the heart. These beneficial actions were exerted through the downregulation of pro-inflammatory cytokines (mainly TNF-α and IL-6) and the upregulation of anti-inflammatory cytokines (mainly IL-10 and TGF-β) via inhibition of the LPS-MyD88 pathway. However, this was a small-scale monocentric study of 24 mice, and these results have not been further reproduced yet.
- **Τ3** and Τ4 significantly regulate tissue development, angiogenesis, and mitochondrial biogenesis, partly via facilitation of tissue adaptation to hypoxia through the p38 MAPK and Akt [248] pathways. Of note, initial low T3 levels are frequent [264] and have been associated with worse outcomes in sepsis [265]. In a mouse model of CLP-induced peritonitis, early T3 administration was associated with reduced lactate and attenuated hypoxia in heart and liver specimens [250]. Furthermore, T3 was recently reported to be beneficial in a murine SICM model via improved calcium homeostasis through phospholamban downregulation [249]. Interestingly, these promising preclinical findings were also translated into a double-blind RCT including 95 severely ill patients with septic shock. In patients with low T3 and T4, oral T3 at high doses for 4 days was associated with reduced mortality, shorter time on mechanical ventilation, and attenuated inflammatory response [251]. It should be highlighted, however, that patients with isolated low T3 presented higher mortality rates. Positive results have also been reported in a small RCT of 52 patients with acute MI, where T3 improved myocardial systolic function and post-infarction remodeling [266]. Nonetheless, both RCTs were exploratory phase II studies with small samples, and large-scale confirmatory studies are needed.
- **Ginsenoside Rc** (ginseng isolate) was also recently investigated in mice with SICM [252], where it attenuated myocardial injury via inhibition of macrophage activation. The authors found that this anti-inflammatory action was exhibited via downregulation of the Signal transducer and activator of transcription 3 (STAT3)/forkhead box O 3a (FoxO3a) pathway and upregulation of Sirtuin1 (Sirt1). Nonetheless, these results arise from only 15 mice, and echocardiography was performed in the first 24 h with no follow-up measurements. Externally validated and large-scale animal studies with longer follow-ups are needed.
- **α-Ketoglutarate** was also associated with improved histological markers in a small-scale study of 32 male mice with SICM [253] via improvement of mitochondrial function (increased mitophagy and mitochondrial fission) and reduced myocardial apoptosis. The main limitation of the present study was the limited follow-up time and the inability to elucidate the molecular pathways involved in these beneficial effects.
- Mei et al. tested the gasmerdin-D inhibitor Y2 (**GI-Y2**) in mice with CLP- or LPS-induced sepsis [254]. In this SICM model, GI-Y2 attenuated myocardial injury via direct binding to gasmerdin-D, leading to reduced production of cytokines and adhesion molecules as well as attenuation of the macrophage pyroptosis by LPS/nigericin. Additionally, gasmerdin-D blockage inside the macrophages’ mitochondria reduced mitochondrial damage and improved mitochondrial function. Nonetheless, the direct effect of GI-Y2 was only tested in macrophages with unclear actions in cardiomyocytes. Furthermore, the interactions between macrophages and cardiomyocyte were studied in vitro, outside the complex in vivo environment.
- Previous reports have suggested the cardioprotective effects of **sodium octanoate** in mice after ΜΙ through the expression of antioxidants in genes and inhibition of myocardial apoptosis [267]. Based on these results, Lin et al. used sodium octanoate in a murine model of LPS-induced sepsis [255]. Interestingly, the authors found that it exhibited beneficial actions through the inhibition of G protein-coupled receptor 84 (GPR84), leading to antioxidant and anti-inflammatory effects. This was also coupled with improved energy metabolism via increased acetyl-CoA synthesis and upregulation of gene expression related to fatty acid oxidation. Potential limitations were that the mice used to study GPR84 presented with global and not heart-specific GPR84 deficiency, that the improvements in energy metabolism were indirectly evaluated, and that several observed epigenetic modifications were not further explored.
- Gene therapies are also being explored to facilitate targeted drug delivery. In a recent study of LPS-induced sepsis in mice, four hub genes (*Itgb1*, *Il1b*, *Rac2*, *Vegfa*) were identified as candidate therapeutic targets [256]. Based on these results, the authors performed an additional investigatory analysis using the Connectivity Map database, where they identified KU-0063794 and dasatinib as candidate compounds, with several other miRNAs serving as potential therapeutic and/or diagnostic targets. Nonetheless, this was just a hypothesis-generating study with the limitation of inadequate experimental verification of identified genes, whose mechanism should be elucidated in future research.

## 6. Conclusions

Sepsis is associated with profound pathophysiological disturbances and organ damage, including SICM. Several pathophysiological axes contribute to SICM, including inflammation, vasodilation, oxidative stress, ANS imbalance, blunted response to catecholamines, calcium mishandling, mitochondrial dysfunction, metabolic reprogramming, and disturbed endothelial/microcirculatory function. Cardiac arrhythmias frequently complicate sepsis, with AF being the most prevalent. Inflammation, electrolyte imbalances, myocardial ischemia, QT prolongation/dispersion, SNS overactivation, calcium mishandling, and fever-induced arrhythmogenesis in Brugada patients constitute the pathophysiological basis of sepsis-induced arrhythmias. Beyond established therapeutic approaches, several novel molecules are currently investigated, mainly in preclinical murine models. Future research should aim to establish a standardized SICM definition, translate emerging therapeutics into clinical practice, identify novel molecular targets, and implement personalized, phenotype-driven interventions.

## Figures and Tables

**Table 1:** Prevalence and definition of SICM across different studies.

First Author, Year	Estimation-Diagnostic Criteria	Prevalence
Endo T, 2013 [9]	LVEF < 50% (TTE)	23/93 (25%) after 24 h
Orde SR, 2014 [10]	RV GLS > −21% LV GLS > −17% (TTE)	60 patients analyzed: 72% RV dysfunction 69% LV dysfunction 50% LV and RV dysfunction
Lanspa MJ, 2015 [11]	LV GLS > −17% (TTE)	41/68 (60%) after 6 h
De Geer L, 2015 [12]	LV GLS > −15% +/− LVEF < 50% E/é > 15 and/or é < 0.08 m/s (TTE)	31/44 (70%) after 24 h
Dalla K, 2015 [13]	LV GLS > −15% RV GLS > −19%	17/34 (50%) after 48 h
Sato R, 2016 [14]	LVEF < 50% and a ≥10% decrease compared to the baseline LVEF (TTE)	29/210 (14%) after 24 h
Jayaprakash N, 2018 [15]	LVEF < 50% (TTE), cTnT > 0.01 ng/mL or NT-pro-BNP > 500 pg/mL in first 24 h ICU for diagnosis of myocardial dysfunction or LVEF < 50% for myocardial depression	169/578 (29%) myocardial dysfunction and 23/578 (4%) myocardial depression in 24 h.
Jeong HS, 2018 [16]	LVEF < 50% and/or ≥10% decrease from baseline LVEF (TTE)	25/325 (8%) after 24 h
Narváez I, 2018 [17]	LV systolic dysfunction (LVEF < 50%) attributable to sepsis, excluding patients with previous heart disease, associated or not to RV systolic dysfunction or LV diastolic dysfunction	13/57 (23%) in 24 h
Cheng MM, 2019 [18]	Sepsis + LVEF ≤ 50% or LVEDD > 50 mm-TTE) or ≥2 of: cTnI > 3x ULN, NT-ProBNP > 3x ULN, low cardiac output manifestations, requirement for inotropes	36/88 (41%) in 24 h
Lu NF, 2019 [19]	Sepsis + one of: LV-Sm < 8 cm/s or LVEF < 50%, RV-Sm < 12 cm/s, E/e′ > 15 or e′ < 8 cm/s, with no history of heart disease	48/93 (52%) over the course of 7 days since admission
Chen FC, 2020 [20]	LVEF < 50% (TTE) or need for inotropes (milrinone/dobutamine) or vasopressors + biomarkers (h-FABP, MPO, cTnI)	70/147 (48%) in 24 h
Wang L, 2021 [21]	LVEF < 50% (TTE), hs-TnI > 0.78 ng/mL or NT-proBNP > 500 pg/mL in first 24 h ICU (Mayo Clinic criteria)	35/75 (47%) in 24 h
Tucker RV, 2022 [22]	LVEF ≤ 55% or decrease from baseline resulting in recategorization of patients from a higher LVEF category to a lower using thresholds: normal: >55%; mildly reduced: 41–55%; moderately reduced: 30–40%; severely reduced: <30% (TTE)	9/110 (8%) in 24 h
Cutuli SL, 2023 [23]	New-onset cardiac dysfunction unrelated to ischemia + at least 1 of: LVSD (LVEF <45%), LVDD (lateral e′ < 8 cm/s), RVD (TAPSE < 16 mm with systolic pulmonary arterial pressure < 35 mm Hg)-using TTE	60/148 (41%)
Zhang J, 2023 [24]	Septic patients with LVEF < 50% (TTE)	22/79 (28%) in 24 h
Hendrickson KW, 2024 [25]	Septic shock patients with LVEF ≤ 55% or decrease in LVEF ≥ 10% from baseline (TTE)	207/1229 (17%) in 72 h
Chang X, 2024 [26]	Sepsis + no pre-existing heart conditions + LVEF < 50% (TTE)	56/270 (21%) in 24 h
Yang X, 2025 [27]	Acute reversible cardiac function changes within 5 days ICU + global or unilateral ventricular dysfunction [LVEF < 50% OR (TRV > 2.8 m/s, LAVi > 34 mL/m^2^, septal e′ wave < 7 cm/s or lateral e′ < 10 cm/s, and E/e′ ratio >13-lateral or >15-septal) OR (RV TAPSE < 16 mm/s or sTDI < 10 cm/s)] + exclusion of myocardial ischemia (TTE)	110/181 (61%) after 5 days since admission
Zhou YT, 2025 [28]	Infection + organ dysfunction + elevated troponin I + ≥ 1 of: myoglobin, CK-MB, α-HBDH (SAMI diagnostic criteria)	316/517 (61%) in 24 h

Abbreviations: SICM, sepsis-induced cardiomyopathy; LV, left ventricle; LVDD, left ventricular diastolic dysfunction; LVEDD, left ventricular end diastolic diameter; LVEF, left ventricular ejection fraction; GLS, global longitudinal strain; LVSD, left ventricular systolic dysfunction; NT-pro-BNP, N-terminal pro-brain natriuretic peptide; cTn, cardiac troponin; RV, right ventricle; ULN, upper limit of normal; TRV, tricuspid regurgitation velocity; LAVi, left atrial volume index; RVD, right ventricular dysfunction; TAPSE, tricuspid annular plane systolic excursion; MPO, myeloperoxidase; h-FABP, heart-type fatty acid binding protein; ICU, intensive care unit; CK-MB, creatine kinase-MB; α-HBDH, α-Hydroxybutyrate Dehydrogenase; SAMI, sepsis-associated myocardial injury; TTE, transthoracic echocardiography.

**Table 2:** Novel therapeutic agents in SICM.

Therapeutic Agent	Mechanism	Evidence
Vitamin C	Antioxidant properties → protection from oxidative stress [230] Anti-inflammatory [230,231]	Small trials with relatively few patients [232]; potential benefit of early administration (<2 h) in patients with marked inflammatory response (observational evidence) [233]
Melatonin	↓ Mst1 expression → ↓ mitochondrial apoptosis [234] JAK/STAT3 pathway regulation and ↓ hypoxia-inducible factor [235,236,237] ↑ iNOS activity → vasodilation [238] Activation of PI3K/Akt signaling [239]	Preclinical SICM models in mice with promising efficacy and safety; lack of evidence in humans (including route and dose of administration), though with relatively high anticipated safety
Engineered exosomes	Vehicles facilitating targeted molecular transportation Direct drug delivery (e.g., MiR21 to cardiomyocytes) [240] Inflammatory response regulation (potent anti-inflammatory actions) [241] Enhancement of protective and reparatory pathways → ↑ cell survival [242,243]	Preclinical mouse models in SICM; small-scale studies have examined exosomes in humans in various clinical conditions with promising results [244,245,246], though large-scale studies are lacking
Schistosoma japonicum-produced cystatin (Sj-Cys)	LPS-MyD88 pathway inhibition → anti-inflammatory action: Downregulation of pro-inflammatory cytokines (TNF-α, IL-6) Upregulation of anti-inflammatory cytokines (IL-10, TGF-β)	CLP model of sepsis in mice [247]
Triiodothyronine (T3)	Regulation of thyroid hormone homeostasis P38 MAPK and Akt pathways → enhanced adaptation to hypoxia [248] Phospholamban downregulation → regulation of calcium homeostasis [249]	Preclinical models in mice with SICM [250]; promising results in an RCT of patients with septic shock (not specifically examined in SICM) [251]
Ginsenoside Rc (substance isolated from ginseng)	STAT3/FoxO3a/Sirt1 pathway modulation → anti-inflammatory action via inhibition of macrophage activation	Mouse model of SICM [252]
a-Ketoglutarate	Increased mitochondrial mitophagy and fission → improved mitochondrial function Reduced myocardial apoptosis	Mouse model of SICM [253]
Gasmerdin-D inhibitor Y2 (GI-Y2)	GI-Y2 binding and blocking gasmerdin-D ↓ production of adhesion molecules ↓ production of pro-inflammatory cytokines ↓ macrophage pyroptosis (cell death) induced from LPS/nigericin	Mouse model of CLP- or LPS-induced SICM [254]
Sodium octanoate (hydrophilic product of saturated fatty acid)	GPR84 blockage → antioxidant and anti-inflammatory actions ↑ acetyl-CoA synthesis and upregulation of genes related to fatty acid oxidation → improved energy metabolism	Mouse model of LPS-induced SICM [255]
Gene-targeted therapies	Identification of genes (*Itgb1*, *Il1b*, *Rac2* and *Vegfa*) encoding molecular mediators in SICM Identification of miRNAs implicated in SICM Targeted drug development and improved diagnosis	Gene identification in a mouse model of LPS-induced SICM [256]

Abbreviations: SICM, sepsis-induced cardiomyopathy; JAK, Janus kinase; STAT3, signal transducer and activator of transcription proteins 3; Mst1, Macrophage-stimulating 1; iNOS, inducible nitric oxide; PI3K, phosphatidylinositol 3–kinase; MiR21, micro-ARN 21; LPS, lipopolysaccharide; TNF-α, tumor necrosis factor-α; IL, interleukin; TGF, tumor growth factor; MAPK, mitogen-activated protein kinase; RCT, randomized controlled trial; FoxO3a, forkhead box O 3a; Sirt1, Sirtuin1; GPR84, G protein-coupled receptor 84; coA, coenzyme A.

## Data Availability

All data extracted and analyzed in this review are found within the main text and accompanying tables.
